# Seasonal Prey Abundance and Food Plasticity of the Vulnerable Snow Leopard (*Panthera uncia*) in the Lapchi Valley, Nepal Himalayas

**DOI:** 10.3390/ani13203182

**Published:** 2023-10-12

**Authors:** Narayan Prasad Koju, Kamal Raj Gosai, Bijay Bashyal, Reena Byanju, Arati Shrestha, Paul Buzzard, Willian Bill Beisch, Laxman Khanal

**Affiliations:** 1 Center for Post Graduate Studies, Nepal Engineering College, Pokhara University, Lekhnath 44800, Nepal; narayanpk@nec.edu.np; 2 Department of Psychology, University of Washington, Guthrie Hall (GTH), Seattle, WA 98105, USA; 3 Trichandra Multiple Campus, Tribhuvan University, Kathmandu 44613, Nepal; kamal.gosai@trc.tu.edu.np; 4 Central Department of Environmental Science, Tribhuvan University, Kathmandu 44613, Nepal; bijay.bashyal049@gmail.com; 5 Patan Multiple Campus, Tribhuvan University, Kathmandu 44613, Nepal; reenabyanju1@gmail.com; 6 Department of Environment, Ministry of Forest and Environment, Kathmandu 44600, Nepal; arati191@gmail.com; 7 China Exploration and Research Society, Hong Kong, China; p_buzzard@yahoo.com (P.B.); billbleisch@cers.org.hk (W.B.B.); 8 Washtenaw County Conservation District, Ann Arbor, MI 48103, USA; 9 Central Department of Zoology, Institute of Science and Technology, Tribhuvan University, Kathmandu 44613, Nepal

**Keywords:** apex predator, flagship species, micro-histology, niche overlap, prey preference

## Abstract

**Simple Summary:**

The snow leopard is an apex predator, residing in mountain ecosystems in Asia. This study was conducted in the Lapchi Valley of the Nepal Himalayas between November 2021 and March 2023 to investigate the seasonal variations in its prey availability and selectivity. Through camera traps and scat analysis, we identified the blue sheep, Himalayan musk deer, domestic horse, and sheep as key prey species for snow leopards. Snow leopards exhibited dietary diversity, consuming eleven prey species, with blue sheep as their primary wild prey and horses as preferred livestock. Seasonal variation in food preference was noted, where small mammals filled the nutritional requirements during winter’s prey scarcity. The study recommends genetic tools for further diet analysis and stresses the importance of transboundary research and population assessments in shaping effective conservation strategies.

**Abstract:**

Conservation strategies for apex predators, like the snow leopard (*Panthera uncia*), depend on a robust understanding of their dietary preferences, prey abundance, and adaptability to changing ecological conditions. To address these critical conservation concerns, this study presents a comprehensive evidence on prey availability and preferences for snow leopards in the Lapchi Valley in the Nepal Himalayas from November 2021 to March 2023. Field data were collected through the installation of twenty-six camera traps at 16 strategically chosen locations, resulting in the recording of 1228 events of 19 mammalian species, including domesticated livestock. Simultaneously, the collection of twenty snow leopard scat samples over 3800 m above sea level allowed for a detailed dietary analysis. Photo capture rate index and biomass composition analysis were carried out and seasonal prey availability and consumption were statistically analyzed. A total of 16 potential prey species for the snow leopard were documented during the study period. Himalayan musk deer (*Moschus leucogaster*) was the most abundant prey species, but infrequent in the diet suggesting that are not the best bet prey for the snow leopards. Snow leopards were found to exhibit a diverse diet, consuming eleven prey species, with blue sheep (*Pseudois nayaur*) being their most consumed wild prey and horses as their preferred livestock. The Pianka’s index of dietary niche overlap between the summer and winter seasons were 0.576, suggesting a pronounced seasonal variation in food preference corroborating with the prey availability. The scarcity of larger preys in winter is compensated by small and meso-mammals in the diet, highlighting the snow leopard’s capacity for dietary plasticity in response to the variation in resource availability. This research suggests for the utilization of genetic tools to further explore snow leopard diet composition. Additionally, understanding transboundary movements and conducting population assessments will be imperative for the formulation of effective conservation strategies.

## 1. Introduction

The charismatic snow leopard (*Panthera uncia*) is the flagship species in the high mountains of Asia, categorized as globally vulnerable in the IUCN Red List of Threatened Species [1,2]. Snow leopards have a wide distribution across 12 central Asian countries, but are not common. In Nepal, they are distributed along the northern frontier [3] and the country is thought to harbor 300–500 snow leopards, making Nepal a key range country [4]. Snow leopards in Nepal have been reported in Kangchenjunga Conservation Area (CA), Manaslu CA, Annapurna CA, Makalu Barun National Park (NP), Sagarmatha NP, Shey Phoksundo NP, and Dhorpatan Hunting Reserve [2,3,4,5,6], Langtang NP [7], and Api Nampa CA [8]. The WWF [9] has estimated that 39 snow leopards inhabit the Eastern Himalayas of Nepal (stretching from Kanchanjunga CA to Langtang NP). Though the potential habitats for snow leopards is contiguous from east to west along the Himalayan range of Nepal, the photographic evidence of snow leopards in Gaurishankar CA was only recently provided by our team [10]. The snow leopard is the apex predator of the Himalayas, an indicator of a healthy mountain ecosystem [11], and it plays a key role in maintaining biodiversity in the ecosystem through predation dynamics and trophic cascades [12].

Snow leopard populations are thought to be declining across most of its range [13,14] due to various types of threats like habitat degradation and fragmentation [15,16], poaching for pelts and bones, killings of snow leopards in retribution for predation on livestock, and reduction of natural prey populations due to illegal hunting, as well as competition from livestock; all of these present threats to snow leopard population viability [3,13]. Detailed information on snow leopard diet and seasonal food plasticity is thus essential for effective conservation management of snow leopards.

Snow leopards are among the opportunistic predators that exploit a wide range of prey species [17], notably blue sheep, markhor, urial, ibex, goats [13,14] and unidentified birds, and a wide variety of medium and small sized mammals, such as marmots, pikas and other rodents [18,19]. In several areas of Nepal, Shrestha et al. [20] have shown seasonal differences for snow leopards in terms of prey items and consumption of livestock. Similarly, Chetri et al. [21] concluded that snow leopards significantly depredated livestock species like horses and goats. The diet composition and analysis, along with prey abundance in their natural habitat, can help to reveal the plasticity of a predator and its ability to potentially adjust its diet in response to the availability of different prey items. An understanding of the snow leopard’s diet is important in order to explain other aspects of its ecology and to design and implement conservation programmes, especially when an animal is facing conservation challenges and is as secretive as the snow leopard [5,13,18]. This study aimed to explore the seasonal prey abundance and food plasticity of snow leopards in the Lapchi Valley, Gaurishankar CA in central Nepal for future conservation and wildlife management.

## 2. Materials and Methods

### 2.1. Study Area

This study was conducted in Lapchi Valley, which lies in the Gaurishankar Conservation Area (GCA) in central Nepal Himalayas, one of the world’s most climatically diverse landscapes. The GCA is a key site for snow leopards since it connects the larger contiguous Tibetan Plateau to the north, Langtang NP to the west, and Sagarmatha NP to the east [11,19,22,23].

Until recently, Lapchi Valley was not connected by modern transportation and communication networks. Its remoteness was responsible for it being among the holiest places for Buddhists since long ago. Lapchi Valley comprises sub-tropical to nival bio-climatic zones with 16 major vegetation types [22,23]. Recorded faunal diversity includes 235 bird species, 77 mammal, 16 fish, 14 snake, 10 amphibian and 8 lizard species [10,24,25]. Musk deer, Himalayan bear, Chinese pangolin, Assamese macaque, snow leopard, and leopard cat are some of the nationally threatened species living in the GCA [23,26]. Major precipitation in the area includes rain during the summer monsoon from June to August, and snow in winter from January to March.

Lapchi Valley is located at the foot of the Lapchi Khang mountain range. This is an important pilgrimage destination for Tibetan Buddhists and the valley is known for the meditation caves of the most famous Tibetan saint and poet, Jetsun Milarepa. The caves surround the main monastery of Lapchi, ‘the ChöraGephel Ling.’ Because of this special religious status, humans and wildlife (including nature and the snow leopard) live harmoniously in Lapchi Valley. However, the local pastoralists often suffer from the predation of livestock by snow leopards and the Himalayan black bear [27].

### 2.2. Camera Trap Surveys

The study area was divided into 16 grids of 2 km × 2 km and, among them, camera traps (CTs) were installed in 14 randomly selected grids. A total of 26 (Initially 18 then 8 additional camera traps replaced after they lost or malfunctioned during study period) Bushnell Trophy Cameras (Model #119537C) were set in hybrid mode from November 2021 to March 2023, so as to take both photographs and videos simultaneously. Camera traps were set 30–40 cm above the ground depending on slope of the land [28,29]. Camera sensors were generally faced away from direct sun rays to avoid unnecessary pictures triggered by light following standard protocol used by Rovero et al. [30]. A one-second trigger time was used between trigger events. The cameras were run for 24 h each day, and used infrared Light Emitting Diodes (LEDs) to record night images.

**Figure 1:**
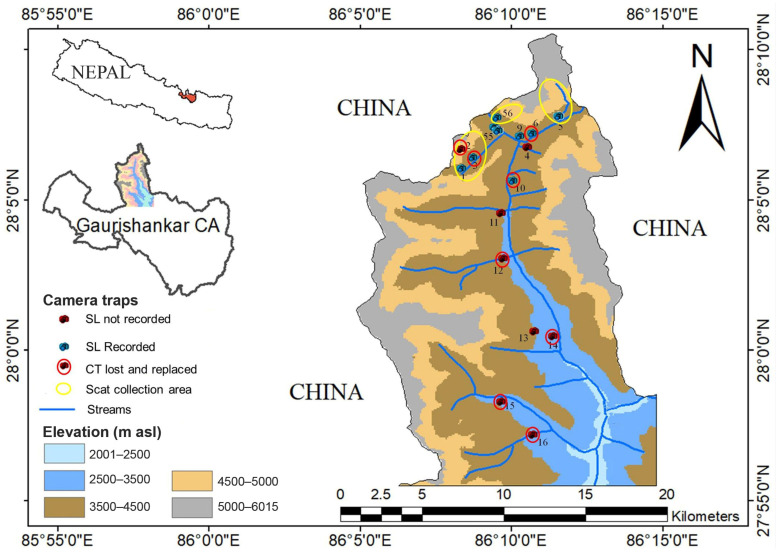
Map showing locations of stations where camera traps were installed in the study area (SL: snow leopards, CT: camera traps).

Among them, at two grids, camera traps were installed at a distance of 1 km apart on either side of the river (Cam 55 and cam 56; Cam 4 and Cam 9) and a pair of camera traps were installed in two locations (Cam 3 and Cam 6) (see Figure 1). During the study period, eight camera traps from four locations were lost, damaged or malfunctioned. The total number of camera trap (CT) days was 3023 with the total events during the study period being 1228. The sites were visited four times to download data, change batteries, and to collect scats/fecal samples of snow leopards. Prey species recorded in the camera traps were categorized into four categories: livestock (horse, and yak), wild ungulates (musk deer, blue sheep, Himalayan tahr, Himalayan goral, and serow), meso-mammals (Assamese macaque, red fox, and yellow- throated marten) and small mammals (pika, weasel and stone marten) following Khatoon et al. [31].

### 2.3. Scat Collection and Laboratory Analysis

Scat samples of the snow leopards were collected in accordance with established protocols developed by Panthera and the Snow Leopard Trust [3] and PAWS [32]. The scat samples were collected over the course of two seasons, winter and summer, to assess and analyze the seasonal diversity and plasticity of the snow leopard’s diet. We implemented a robust seasonal categorization system, dividing the year into two distinct periods: summer, spanning 16 April to 15 November; and winter, from 14 November to 15 April. This categorization was based on empirical observations of the movement patterns of yaks and other livestock as they moved from high-altitude pastures to lower-elevation villages. This livestock migration served as a reliable and practical indicator of the changing seasons in our study area, ensuring the accuracy and relevance of our seasonal distinctions. A total of 20 scat samples, ten in each season, were collected throughout a sixteen-month period. All the scat samples were found at altitudes above 3800 m above sea level (asl), exclusively within alpine areas. These specific sites were chosen due to confirmed records of snow leopard presence and absence of the common leopard, as indicated by camera trap data in this and previous study periods.

A small portion of the scat was carefully collected and placed in a plastic tube for subsequent micro histological analysis. Upon encountering a snow leopard scat, a careful procedure was followed to ensure the preservation of valuable information while minimizing any disruption to the predator’s regular movements and territorial marking. To maintain ecological integrity and respect for the natural behavior of these elusive predators, a significant portion of the scat was deliberately left undisturbed in the field [13]. The micro histological analysis of scats was performed in the laboratory of Center for Postgraduate Studies, Nepal Engineering College, Prayagpokhari, Lalitpur; Patan Multiple Campus, Lalitpur and Central Department of Environmental Science Tribhuvan University, Kathmandu, Nepal. Diet composition was analyzed following the protocol of Oli [33]. Scat samples were systematically collected during multiple time points to capture seasonal variations and dietary trends, with collection dates in October 2021, April 2022, August 2022, December 2022, and March 2023. All the collected scat samples underwent a thorough washing process using tap water, employing a fine mesh sieve to separate and retain items such as bones and teeth for subsequent prey species identification. Following this initial wash, the samples were subjected to further cleaning in carbon tetrachloride (CCl_4_) and then dried between absorbent paper. Subsequently, the samples were carefully oven-dried at a controlled temperature of approximately 60 °C. A systematic approach was taken to select representative prey samples for analysis. Specifically, twenty hairs from potential prey were randomly sampled from each scat. To ensure objectivity and randomness, an A4 sheet of paper was divided into twenty equal 1 × 1-inch boxes, each designated with a unique color. Dried hairs were then dispersed randomly on the paper, and the identification of hairs on one box randomly chosen was performed to ensure unbiased and comprehensive prey analysis in accordance with the guidelines outlined by Mukherjee et al. [34].

Each hair was mounted with DPX (Dibutylphthalate Polystyrene Xylene) on a slide and was covered with a cover slip, observed in the light via a microscope with magnification of 400×, and photos were thus taken. Prey species were identified by examining the structure of the medulla and comparing with reference slides of hair samples [33,35,36,37]. A reference slide of wild animals and domesticated livestock were made from the study area (Supplementary Figure S1), and unavailable hair samples of possible prey species were compared with published articles [18,20,31,38].

### 2.4. Data Analysis

For the analysis of camera trap data Photo Capture Rate Index (PCRI) was used. Consecutive images of individuals of the same species separated by intervals of 30 min or more between them, different individuals of the same or different species in successive photographs, and non-consecutive photos of individuals of the same species at the same site were considered independent. Blank images and images from which species that could not be identified were not included in the analysis. PCRI was used as an index of species abundance at the locality [12]. This approach has provided valuable insights into the relative abundance and presence of various species through camera trap data analysis [12,39,40].

Prey consumption calculations based on hair numbers are known to be biased, so we used the consumption of prey based on the biomass consumption for the overall year, based on Ackerman et al. [41]. The frequency of occurrence (F) of the various prey species (the proportion of all the scats in which a prey species was detected) was determined from the scat content. This direct approach is well known to be biased [31,42,43]. Therefore, used Ackerman’s Equation:

B = 1.98 + 0.035A,

where B is the mass of prey (kg) per leopard scat sample and ‘A’ is the average mass of an individual of a particular prey species [41]. On this basis, we calculated total biomass (D) and the number of prey individuals € consumed by snow leopards. The average weight of the respective wild prey species used was based on Shrestha et al. [44], Amin et al. [45], and Karanth and Sunquist [46]. The average weight of yak used was 80 kg, and for horse, 125 kg, based on local herders’ reports of the sizes of horse and yak they lost to snow leopards during the study period.

The estimated percentage of prey consumed was calculated using formula

E = Percentage consumption (B × C/∑ [B × C] × 100).

where

B = Estimated weight of prey consumed per scat (B = 1.98 + 0.035 × A) [41].

C = Number of scats in which prey species were identified.

Then, Biomass consumed D = B × C.

Seasonal variations in the number of preys obtained from camera trap data and the variations in number of hairs in the scats of snow leopards were tested for the statistical significance using χ^2^-test. The prey species were categorized into four major categories- livestock, wild ungulates, meso-mammals and small mammals.

The niche breadths of snow leopards in the study seasons was calculated by standardized Levin’s measure of niche breadth (B).

$$B=\frac{1}{\sum p_{i}^{2}}\;$$

$$B\prime =\frac{B-1}{n-1}$$

where B = Levin’s measure of niche breadth, B’ = Standardized niche breadth, p_i_ = Proportion of resource i in the diet, and n = total number of possible resource states.

Additionally, the dietary niche overlap between the summer and winter seasons was tested by Pianka’s measure of niche overlap [47] in ‘EcoSimR’ package [48] in R statistical software version 4.3.1 [49], using the data on biomass of preys consumed.

$$O_{\mathrm{sw}}=\frac{\sum_{i}^{n}p_{\mathrm{is}}p_{\mathrm{iw}}}{\sqrt{\sum_{i}^{n}p_{\mathrm{is}}^{2}\;\sum_{i}^{n}p_{\mathrm{iw}}^{2}}}$$

where O_sw_ = Pianka’s measure of dietary niche overlap between the summer and winter seasons

p_is_ = Proportion of resource I is of the total resources used in the summer season

p_iw_ = Proportion of resource I is of the total resources used in the winter season

n = total number of resource states.

## 3. Results

### 3.1. Preys of Snow Leopard in Lapchi Valley

A comprehensive assessment of mammalian species within the study area recorded a total of 19 species, consisting of both domestic livestock and wild mammals. While camera traps successfully documented the majority of these species, some rodent species and the Himalayan marmot were not captured on the cameras but were observed directly during field visits. Among the recorded mammals, the Himalayan musk deer (*Moschus leucogaster*) had the highest frequency of occurrence with 534 events, followed by domestic yak (*Bos mutus*) with 148, and the red fox with 101 capture events. Snow leopards were recorded in 37 events at eight camera stations, while the common leopard and Himalayan black bear (*Ursus thibetanus laniger*) were recorded in 53 and 29 events, respectively. The Himalayan musk deer was the most abundant species with a Photo Capture Rate Index (PCRI) of 24.4. Other notable species included the wild yak (PCRI = 6.03) and Royle’s pika (*Ochotona roylii*) (PCRI = 4.97). In contrast, the PCRI-based abundance for the snow leopard was 1.79, common leopard was 2.97, and the Himalayan black bear was 2.28 (Table 1).

### 3.2. Seasonal Prey Diversity and Abundance in Lapchi Valley

**Figure 2:**
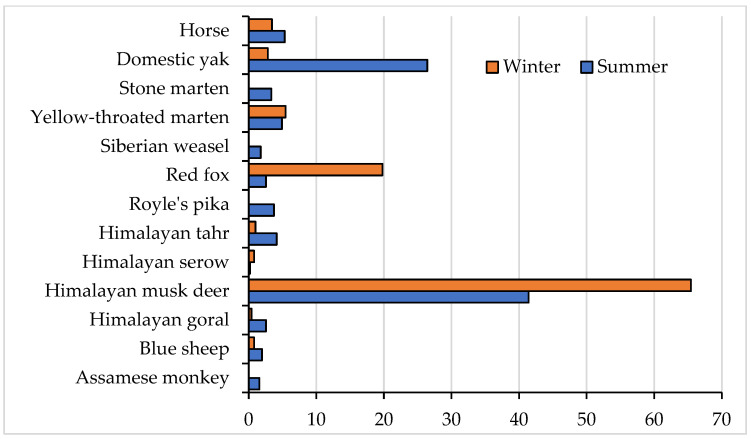
Seasonal prey abundance in the study area based on camera traps data.

A total of 16 (out of 19 recorded in CT) potential prey species for the snow leopard were documented during the study period. Most of these species were observed in both the summer and winter seasons, with the exception of the Himalayan serow, Assamese macaque, Siberian weasel, stone marten and livestock (yaks and horses), which exhibited season-specific patterns. Specifically, the Assamese macaque, Siberian weasel, and stone marten were absent during winter, while Himalayan serow was not recorded in camera traps in the summer. Livestock were brought to lower elevation during winter and thus largely excluded from our camera traps (Figure 2).

The Himalayan musk deer was persistent in the habitat, recording the highest frequency of occurrence in both summer and winter seasons and highlighting its year-round availability as a prey species for snow leopards. While camera traps captured images of the Assamese macaque, Himalayan goral, Himalayan serow, yellow-throated marten, and stone marten, these species were notably absent from the histological analysis of snow leopard scats, indicating potential dietary selections and behavior patterns that provide need or scope for further investigation.

The Chi-square test revealed significant seasonal differences (*p* < 0.05) in the abundance of livestock species, meso-mammal species, consumed wild ungulates, and consumed wild prey species (Table 2). While the abundance of wild ungulate prey species remained relatively consistent throughout the year according to camera trap records, the availability of preferred wild ungulates, including the Himalayan musk deer, Himalayan tahr, and blue sheep, exhibited significant seasonal variations.

### 3.3. Diet Composition of Snow Leopard

Micro-histological analysis of snow leopard scat in Lapchi Valley provided valuable insights into their dietary preferences, revealing the consumption of eleven distinct prey species. Weasel, rodents, pika, Himalayan musk deer, Himalayan tahr, Himalayan marmot, and blue sheep were consumed in both summer and winter seasons. Yak and horse were identified as prey items year-round, while domestic sheep consumption was exclusive to the summer season, and fox hairs were detected only in the summer.

During the winter, blue sheep contributed the most (44%) in the diet of snow leopards, followed by musk deer (20.5%), horse (9%) and yak (6.5%). In contrast, the summer dietary composition displayed variations; the domestic goat constituted the highest proportion (32.5%), followed by horse (25.5%), blue sheep (15.5%), and yak (11.5%). These three livestock species collectively constituted over 60% of the snow leopard’s summer diet. Among wild preys, blue sheep contributed 15.5%, while musk deer accounted for 5.5% of the diet composition during summer (Table 3).

A significant difference (*p* < 0.05) was observed in snow leopards’ food preferences for livestock and meso-mammals between summer and winter seasons (Table 4). Remarkably, the dependency on small mammals and wild ungulate species remained consistent across seasons, highlighting their importance as staple dietary components.

**Figure 3:**
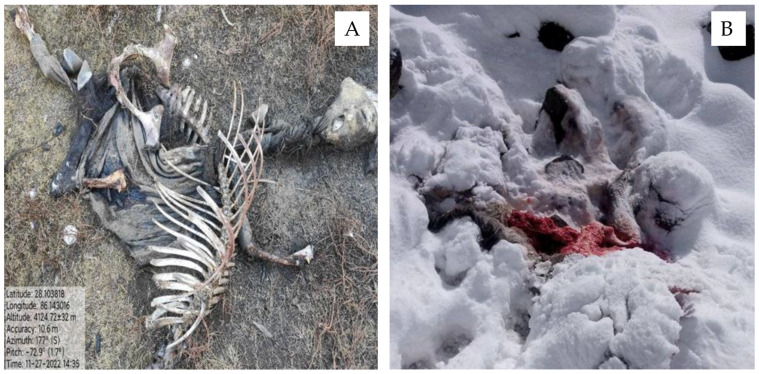
Preys of snow leopards in Lapchi Valley. (**A**) A carcass of a horse in the field at elevation 3924 masl in November, 2022; (**B**) Snow leopard sign with a freshly killed prey at elevation 3842 masl in March 2023 (Photo credit-Renjen Sangrenjen).

During our field visit in November, we noticed the carcass of a horse near CAM2 station (3924 masl). Local herders reported that a horse was killed by a snow leopard in October, and recorded in March 2023 near camera station CAM4 (3842 masl) (Figure 3 and Supplementary Table S3), another horse was killed. These observations corroborated the findings from scat analysis, with evidence of snow leopard predation on horses in October and February, supporting the conclusion that livestock are important food source both in summer and winter.

Among the 20 collected scat samples, three of them exclusively contained the hair of a single species, with two instances being attributed to blue sheep in winter and one to a horse in summer. A more complex dietary spectrum was observed in the remaining scat samples. Eight samples exhibited hair samples from two distinct prey species, while nine samples contained evidence of the presence of more than two prey species. The presence of hair samples of specific prey species varied within the scat samples. Notably, the highest frequency of occurrence was attributed to blue sheep, with seven out of the 20 scat samples containing noticeable hair samples of this particular prey species. Moreover, hair samples of rodents were identified in six samples, whereas domestic goat, pika, musk deer, and yak were encountered in five samples each. Horse and marmot hair were documented in four samples. Intriguingly, domestic sheep consumption was confined to the summer season only. Despite musk deer’s higher abundance recorded via camera traps, only five scat samples (25%) contained musk deer hair, implying a potential lower preference for this wild prey species (Supplementary Table S1).

### 3.4. Biomass of Prey Consumption

Biomass consumption analysis revealed that snow leopards predominantly consumed horses (18.54%), followed by blue sheep (15.47%), yak (13.95%), domestic goats (10.41%), musk deer (8.7%) and rodents, who contributed to the remainder of the consumed biomass. The consumption exhibited notable seasonal differences (Table 5). Domestic animals such as horse (25.74), Yak (19.36) and goats (19.27) constituted the major diet of snow leopards in the summer season. Conversely, wild animals like blue sheep (24.04%), rodents (12.61%), pika (12.61%) and musk deer (11.42) contributed a higher proportion in the winter diet.

Snow leopards in Lapchi Valley had a wider Levin’s niche breadth during the winter (0.638) than in the summer (0.526). The Pianka’s measure of niche overlap between the seasons was 0.576 indicating a higher seasonal plasticity in the diet selection (Supplementary Figure S2).

## 4. Discussion

The sixteen-month camera trap records, direct observation, and microhistological analysis of scats for snow leopards yielded robust insights into the profound seasonal shifts in food availability and preference. Several species, including the Assamese macaque (*Macaca assamensis*) (PCRI = 2.4), Himalayan goral (PCRI = 4.5), and Himalayan serow (PCRI = 0.76) were recorded in the camera traps during the summer season within the study area. However, intriguingly, these species were not part of the snow leopard’s diet. These observations suggest that snow leopards may not venture into elevations below 4000 masl during the summer season, as these three species were found exclusively in such regions but still were not consumed. Notably, the lowest elevation recorded for snow leopards in camera traps during summer was 3800 masl and winter was 3555 masl. Histological analysis of scat revealed the presence of 11 prey species, including three livestock, and two out of these three livestock species being raised within the Lapchi Valley. Himalayan musk deer were the most abundant prey in the area, however, their relative frequency in the scats was low, suggesting the lesser preference of the species by the snow leopard. Interestingly, certain carnivore species, like the red fox and Siberian weasel, were consumed by snow leopards. Additionally, prey species, including domestic sheep and red fox, were conspicuously absent from scat samples collected in the winter. Assamese macaque and stone marten, despite being present in the study area during summer, were never found in the snow leopard’s diet. Similarly, yellow-throated marten and Himalayan serow were consistently present throughout all seasons, but were absent from snow leopard scat.

This statistically significant difference in prey consumption between the seasons underlines the discerning nature of snow leopards in their selection of prey, showcasing their adaptability in accordance with the resource availability. Remarkably, the analysis unveiled consistency in the snow leopards’ reliance on small mammals and wild ungulates across both summer and winter seasons [20]. This implies that these particular prey categories remain pivotal constituents of the snow leopard’s diet, exhibiting resilience in the face of seasonal fluctuations and consistently contributing to the snow leopard’s nutritional requirements. In contrast, both the availability and consumption of meso-mammals and wild ungulates remained relatively stable, indicating that snow leopards adapt their dietary preferences by targeting small mammals in winter when livestock are absent or sparse [31].

Moreover, the presence of domestic sheep in summer scat samples suggested that snow leopards exhibit extensive movement patterns, including transboundary movement between the Lapchi Valley and Tibet in China. This phenomenon highlights the snow leopard’s ability to adapt its food preferences based on the availability of preferred prey species, shifting its diet to fulfill energy requirements during different seasons. These findings suggested substantial food plasticity of snow leopards within the Lapchi Valley ecosystem, revealing remarkable adaptability in response to varying ecological conditions.

A comparative analysis between prey availability estimated from camera trap data and direct observation and the prevalence of various species in the diet based on fecal analysis indicates that apex predators, such as the snow leopard, have a deep influence on ecosystem dynamics, making a comprehensive understanding of their dietary habits and foraging strategies crucial for formulating effective conservation measures [21]. We studied the complex dietary dynamics of snow leopards in the Lapchi Valley, revealing significant shifts in food availability and preference between the seasons. Our findings demonstrate that snow leopards in Lapchi Valley consumed a diverse range of prey, comprising eleven species, encompassing both wild and domesticated mammals. Importantly, the study highlights the major variations in diet preference and availability between the seasons. During the summer, snow leopards exhibited a significant predilection for livestock, including domesticated goats, despite the absence of such animals in the local farming practices within Lapchi Valley. This phenomenon aligns with findings from the remote Phu Valley in northern Nepal, where Wegge et al. [50] obtained reliable information on livestock losses and estimated predator abundance and diet composition from DNA analysis and prey remains in scats, they estimated the annual diet consisted of 42% livestock, which is even higher than in Lapchi Valley. Ale et al. [51] reported blue sheep were the most consumed wild prey for snow leopards in Annapurna CA, which is similar to our findings. Moreover, the contrast in livestock consumption between Lapchi Valley and other regions in the Nepal Himalaya emphasizes the importance of localized conservation strategies.

The study focused on habitat suitability for blue sheep by Jackson et al. [52] assumed that adequate supplementary medium and small-sized prey are available in areas optimal for blue sheep, establishing a direct correlation between blue sheep abundance and food suitability for snow leopards, which concluded that the suitability of food resources for snow leopards is intricately tied to the availability of their primary prey. The comparative study on snow leopards and wolves by Chetri et al. [21] unveiled distinct prey preferences between the two predators. Snow leopards exhibited a significant preference for cliff-dwelling wild ungulates, primarily blue sheep, accounting for 57% of identified material in scat samples. In contrast, wolves displayed a preference for plain-dwelling prey like Tibetan gazelle, kiang, and argali. Notably, both predators demonstrated a lower frequency of livestock depredation than would be expected based on the proportional availability of livestock in the Manaslu CA. However, in contrast, snow leopard dietary habits in the Annapurna CA revealed the consumption of seven species of wild and five species of domestic mammals, alongside unidentified mammals and birds. Blue sheep appeared as the most frequently consumed prey, with Himalayan marmots making an appearance in summer and Royle’s pika in winter.

Lyngdoh et al. [53] shed light on the highly specialized diet of snow leopards in reviewing their diet globally, highlighting the Siberian ibex, blue sheep, Himalayan tahr, argali, and marmots as their primary prey. Snow leopards exhibited a significant preference for prey within the weight range of 36 to 76 kg, with a pronounced affinity for Siberian ibex and blue sheep. Hacker et al. [54] employed next-generation sequencing (NGS) and DNA metabarcoding to analyze snow leopard diets, revealing variations in the most common prey species by country. Markhor was prevalent in Pakistan, Siberian ibex in Mongolia and Kyrgyzstan, and blue sheep in China. Livestock comprised a significant portion of diets, accounting for 31% in Pakistan and 15% in Mongolia, encompassing goats, sheep, bovids, and horses. Similarly, Lu et al. [55], by DNA metabarcoding in the Qinghai-Tibetan Plateau’s Sanjiangyuan region, reported that snow leopards primarily preyed upon wild ungulates, particularly blue sheep, which accounted for a mean of 81.5% of sequences. Livestock constituted 7.62% of their diet, with smaller mammals like marmots, pikas, and mice contributing 10.7%. Interestingly, proportional and total livestock consumption by snow leopards increased linearly with local livestock biomass rather than livestock density, suggesting a complex interplay between wild and domestic prey in Qinghai.

Predation by snow leopards on domestic livestock is an important issue for conservation management. In the Manaslu CA, domestic yaks were more frequently consumed than other livestock types [18]. However, in Lapchi Valley, horse was found to be the primary domesticated animal preyed upon by snow leopards, followed by yaks. The local inhabitants in the Annapurna CA, with an average livestock holding of 26.6 animals per household, reported losses to snow leopards averaging 0.6 to 0.7 animals per household [56]. Unfortunately, these interactions often led to negative attitudes toward snow leopards, with some locals advocating for the complete extermination of the species to mitigate predation problems. These finding highlights the potential for high human–wildlife conflict in areas with elevated livestock densities. In Lapchi Valley, the prey preference paralleled that of China, Pakistan and Mongolia, changing in response to prey species availability.

Snow leopards in northwestern Pakistan were recorded to consume a total of 17 prey species, with a notable preference on large mammals, meso-mammals, and small mammals [31]. Livestock constituted a substantial part of their diet, accounting for 26.4%, with seasonal variations showing higher consumption in summer. Meso-mammals collectively contributed 33.4% to the diet, with palm civets dominating at 16.8% [31]. Mallon et al. [57] also emphasized the prevalence of blue sheep and Siberian ibex in the snow leopard diet, supplemented by other mountain ungulates, domestic livestock, medium- and small-sized mammals, birds, and even invertebrates. Shrestha et al. [20] further explained snow leopard dietary patterns in three Himalayan regions in Nepal, namely Sagarmatha NP, Lower Mustang, and Upper Manang. Their study, conducted from 2014 to 2016, involved genetic confirmation of scats and prey availability assessment via camera traps. The results indicated regional variations in prey preference. This study provides comprehensive insights into the dietary dynamics of snow leopards in the Lapchi Valley. The findings suggest the species’ dietary plasticity, their adaptation to seasonal variations in prey availability, and the need for localized conservation efforts, which account for regional nuances in prey preference and human–wildlife interactions. These insights are pivotal for formulating effective conservation strategies aimed at safeguarding the iconic snow leopard in the unique Himalayan ecosystem.

## 5. Conclusions

This study provides comprehensive insights into the dietary dynamics of snow leopards in the Lapchi Valley. The findings revealed the species’ dietary plasticity, their adaptation to seasonal variations in prey availability, and the need for localized conservation efforts, which account for regional nuances in prey preference and human–wildlife interactions. This comprehensive study of snow leopard dietary habits in the Lapchi Valley has yielded significant insights into the species’ exceptional dietary adaptability in response to seasonal variations in prey availability. It has unveiled a rich prey species base, including both wild and domesticated mammals, with wild prey forming a crucial part of the snow leopard’s diet throughout the year. The noticeable shifts in prey preference, with a distinct dependence on livestock during the summer and a more varied diet in winter, highlight the ability of snow leopard to flexibly adjust to changing ecological conditions. These findings emphasize the paramount importance of adapting conservation strategies to the specific needs and shades of regional snow leopard populations, particularly with regard to their prey preferences and human–wildlife interactions. By doing so, we can better safeguard the future of this iconic and enigmatic apex predator within the distinct Himalayan ecosystem.

## Figures and Tables

**Table 1:** List of mammals and their Photo Capture Rate Index in the Lapchi Valley.

Wildlife Recorded in Cameras	Total Photos	CT Days to Respective CTs	No of Recorded CTs	PCRI Per Recorded CT Base
Assamese macaque (*Macaca assamensis*)	8	333	1	2.4
2. Blue sheep (*Pseudois nayaur*)	14	590	3	2.37
3. Himalayan goral (*Naemorhedus goral*)	15	333	1	4.5
4. Himalayan musk deer (*Moschus leucogaster*)	534	2188	8	24.4
5. Himalayan serow (*Capricornis sumatraensis thar*)	3	393	2	0.76
6. Himalayan tahr (*Hemitragus jemlahicus*)	26	333	1	7.8
7. Royle’s pika (*Ochotona roylii*)	19	382	2	4.97
8. Common leopard (*Panthera pardus*)	53	1780	7	2.97
9. Himalayan black bear (*Ursus thibetanus laniger*)	29	1271	5	2.28
10. Himalayan wolf (*Canis lupus*)	46	1272	5	3.61
11. Leopard cat (*Prionailurus bengalensis*)	57	1628	7	3.5
12. Red fox (*Vulpes vulpes*)	101	2690	11	3.75
13. Siberian weasel (*Mustela sibirica*)	9	901	3	0.99
14. Snow leopard (*Panthera uncia*)	37	2063	8	1.79
15. Yellow-throated marten (*Martes flavigula*)	51	1930	7	2.64
16. Stone marten (*Martes foina*)	17	394	2	4.31
17. Domestic dog (*Canis lupus familiaris*)	1	334	1	0.29
18. Domestic yak (*Bos grunniens*)	148	2451	9	6.03
19. Horse (*Equus caballus*)	44	1621	5	2.71
Total events	1212			82.16
CT days	3023			

**Table 2:** Seasonal variation in camera trap records of prey species of snow leopard in Lapchi Valley.

SN	Prey Type	Summer	Winter	χ^2^	df	*p*
	Livestock	31.75	6.26	4.27	1	0.03
	All wild ungulates	50.29	68.48	7.12	4	0.12
	Wild ungulates, consumed (present in scat)	47.53	67.27	4.53	2	0.001
	Wild ungulate, not consumed	2.76	1.21	1.57	1	0.208
	Meso-mammals, all	9.07	25.25	6.96	2	0.03
	Meso-mammals, not consumed	9.86	5.45	4.02	2	0.133
	Small mammals	8.87	0	NA	NA	NA
	Wild prey consumed only *	88.08	53.84	15.28	4	0.0041

* Note: Based on records of scat analysis. NA: Not available.

**Table 3:** Seasonal diet composition of snow leopard in Lapchi Valley based on the microhistological analysis of scats.

Prey Species Common Name	Winter		Summer	
	Number of Occurrences	% of Prey Species Contribution	Number of Occurrences	% of Prey Species Contribution
Weasel	3	1.5	2	1
2. Rodent	12	6	5	2.5
3. Fox	0	0	2	1
4. Pika	9	4.5	3	1.5
5. Musk deer	41	20.5	11	5.5
6. Himalayan tahr	6	3	2	1
7. Himalayan marmot	9	4.5	2	1
8. Blue sheep	88	44	31	15.5
9. Yak	13	6.5	23	11.5
10. Domestic goat	0	0	65	32.5
11. Horse	18	9	51	25.5
12. Snow leopard	1	0.5	0	0
13. Unknown	0	0	3	1.5

**Table 4:** Statistical analysis of seasonal variation in diet of snow leopard in Lapchi Valley based on number of hairs in scat.

Prey Type	Winter	Summer	χ^2^	df	*p*
Livestock	31	139	25.06	2	0.0001
Wild ungulates	135	44	0.46	2	0.791
Meso-mammals	12	6	5.23	2	0.0001
Small mammals	21	8	0.06	1	0.793

**Table 5:** Estimated biomass consumption (kg) by snow leopard at Lapchi Valley, GCA based on microhistological analysis of scats.

Prey	Average Weight	No of Scat with Respective Prey	Biomass Per scat	Biomass Consumed	Estimated Percentage of Biomass Consumed (E)		
	A	C	B	D	Summer	Winter	Total
Weasel	0.50	3.00	2.00	5.99	2.70	6.34	4.37
2. Rodent	0.20	6.00	1.99	11.92	5.37	12.61	8.70
3. Fox	6.00	1.00	2.19	2.19	2.96	0.00	1.60
4. Pika	0.18	5.00	1.99	9.93	2.68	12.61	7.24
5. Musk deer	12.00	5.00	2.40	12.00	6.48	11.42	8.75
6. Himalayan tahr	36.00	2.00	3.24	6.48	4.37	5.14	4.73
7. Himalayan marmot	4.50	4.00	2.14	8.55	2.89	10.17	6.24
8. Blue sheep	30.00	7.00	3.03	21.21	8.18	24.04	15.47
9. Yak	80.00	4.00	4.78	19.12	19.36	7.58	13.95
10. Domestic goat	25.00	5.00	2.86	14.28	19.27	0.00	10.41
11. Horse	125.00	4.00	6.36	25.42	25.74	10.08	18.54

## Data Availability

The data used in the study will be made available upon the request to the corresponding author.

## Supplementary Materials

The following supporting information can be downloaded at: https://www.mdpi.com/article/10.3390/ani13203182/s1, Figure S1: Micro histological slides of reference hair samples for analysis of snow leopard’s scat at 400× magnification; Figure S2: A plot showing the Pianka’s measure of niche overlap with 1000 simulations using EcoSimR Package in R software; Table S1: Record of hair analysis in scats collected in different seasons; Table S2: List of seasonal prey availability and consumption for snow leopard in Lapchi Valley; Table S3: Tentative location of camera trap installation locations; Table S4: List of mammalian species recorded in respective camera trap.
