# Supplementary material for: Seasonal Prey Abundance and Food Plasticity of the Vulnerable Snow Leopard (Panthera uncia) in the Lapchi Valley, Nepal Himalayas
Source: Animals (Basel). 2023 Oct 12;13(20):3182. doi: 10.3390/ani13203182 (PMC10603713; doi:10.3390/ani13203182)
Supplement: Supplementary file 1 [file animals-13-03182-s001.zip › animals-2652982-supplementary.pdf]

Seasonal Prey Abundance and Food Plasticity of the Vulnerable Snow Leopard (*Panthera uncia*) in the Lapchi Valley, Nepal Himalayas

Narayan Prasad Koju [1,2], Kamal Raj Gosai [3], Bijay Bashyal [4], Reena Byanju [5], Arati Shrestha [6], Paul Buzzard [7,8], Willian Bill Beisch [7], Laxman Khanal [9]*

Supplementary Figures:

Supplementary Figure S1: Micro histological slides of reference hair samples for analysis of snow leopard's scat at 400x magnification

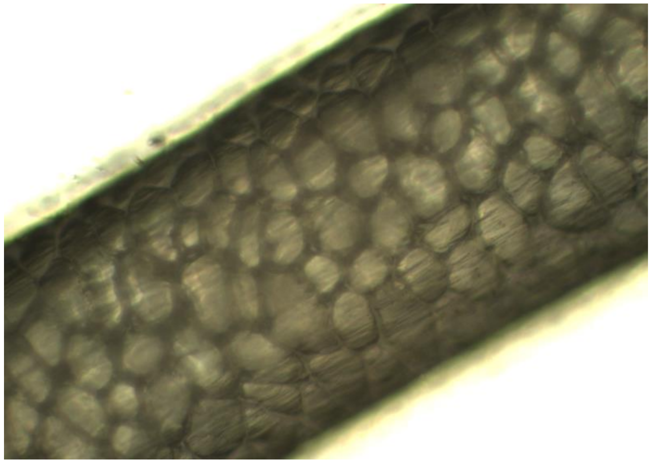

Blue sheep

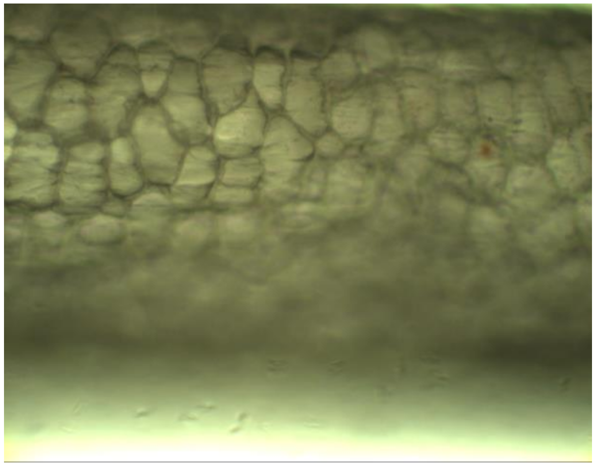

Musk deer

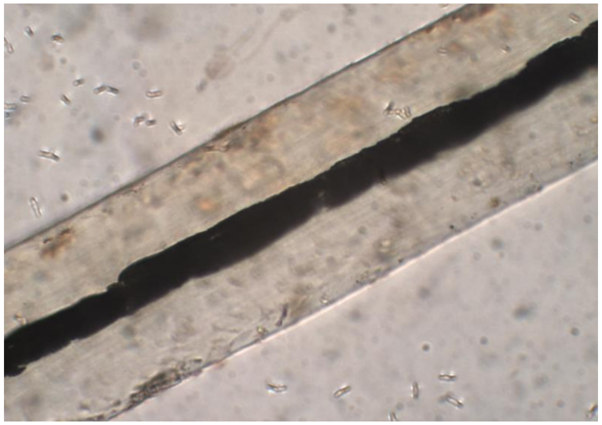
Horse

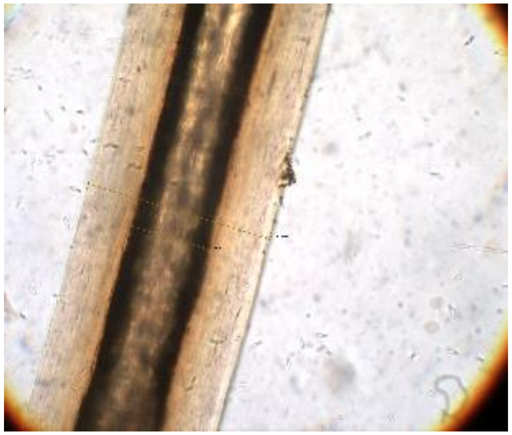
Domestic yak

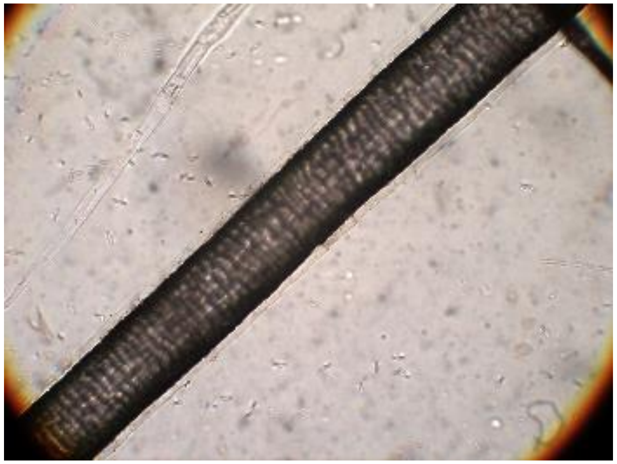
Domestic sheep

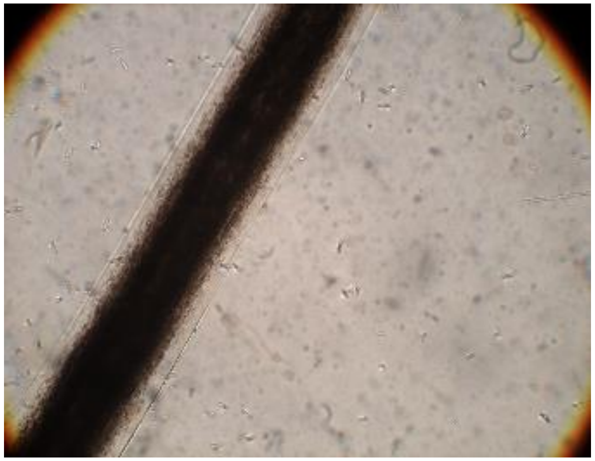
Himalayan Thar

Supplementary Figure S2: A plot showing the Pianka's measure of niche overlap with 1000 simulations using EcoSimR Package in R software

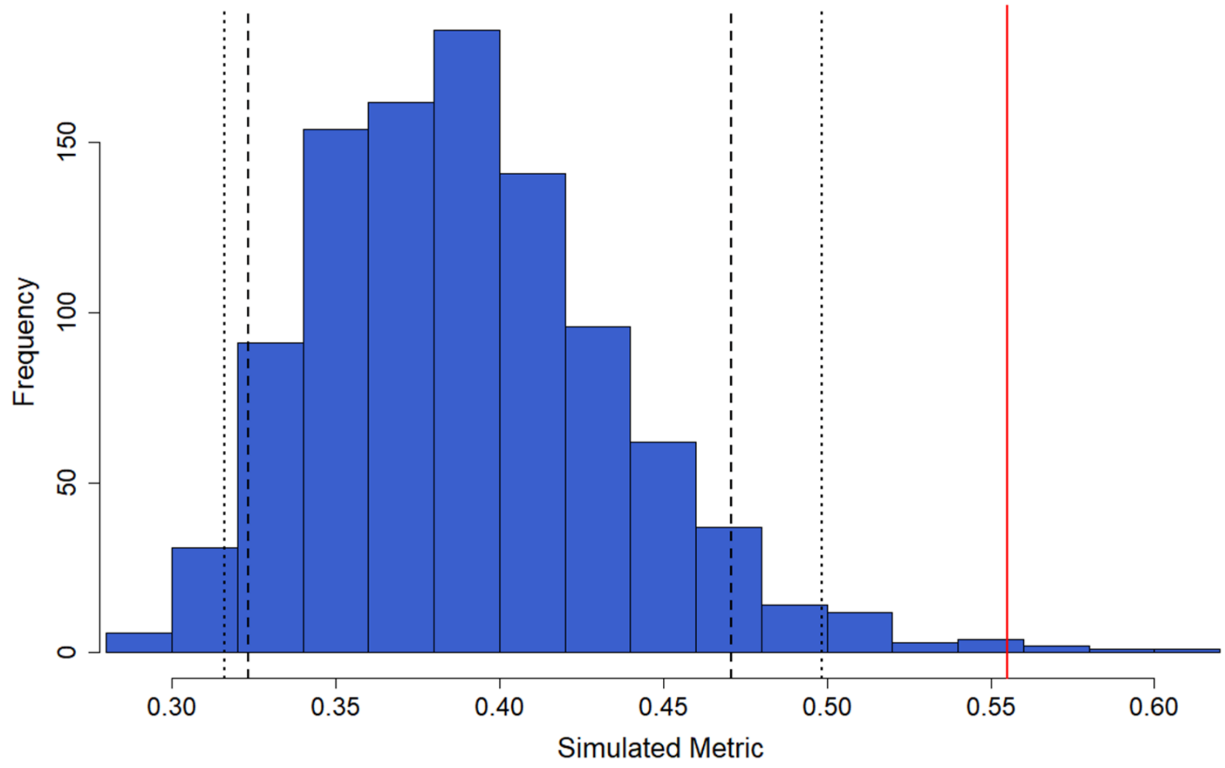

Supplementary tables

Supplementary Table S1: Record of scat hair analysis in scats collected in different seasons

Prey species	Sample name																				P	O
	W1	W2	W3	W4	W5	W6	W7	W8	W9	W10	S1	S2	S3	S4	S5	S6	S7	S8	S9	S10		
	Winter season										Summer season											
1.Weasel					1				2									2			3	7
2. Rodent				1		2		5	4					3	2						**6**	19
3.Fox																				2	1	3
4. Pika					2	2	2	3									3				**5**	13
5.Musk deer							15	12	14		7				4						**5**	54
6.Himalayan Tahr										6								2			2	9
7.Himalayan Marmot	2				4		3									2					4	12
8.Blue sheep		20	20	18		16				14								16	15		**7**	121
1.Yak					13								8			10			5		3	39
2.Domestic goat											13		12		14	8				18	**5**	70
3.Horse	18											20		17			14				4	72
Snow leopard				1																	1	1
Unknown																	3				1	4
Total	20	20	20	20	20	20	20	20	20	20	20	20	20	20	20	20	20	20	20	20	24	420

Note: P= Total scat with presence of prey, O= Total occurrence in of hair in respective scat

Supplementary Table S2: List of seasonal prey availability and consumption for snow leopard in Lapchi Valley

Prey species	***Presence based on CT & DO***		***Consumed based on HAS***	
	Summer	*winter*	*Summer*	*winter*
1. Assamese macaque	□	✗	✗	✗
2. Blue sheep	□	□	□	□
3. Himalayan goral	□	□	✗	✗
4. Himalayan marmot*	□	□	□	□
5. Himalayan musk deer	□	□	□	□
6. Himalayan serow	□	□	✗	✗
7. Himalayan tahr	□	□	□	□
8. Royle's pika	□	✗	□	□
9. Red fox	□	□	□	✗
10. Siberian weasel	□	✗	□	□
11. Yellow-throated marten	□	□	✗	✗
12. Beech marten/stone marten	□	✗	✗	✗
13. Rodents	□	□	□	□
1. Domestic yak	□	□	□	□
2. Horse	□	□	□	□
3. Domestic sheep	✗	✗	□	✗

Note: CT Camera trap, DO Direct observation, HAS Histological analysis of scat, □ presence, ✗ absent

Supplementary Table S3: Tentative location of camera trap installation locations*

SN	Location	Latitude	Longitude	Elevation	References	Status
1.	CAM 55	28.121697°	86.154420°	4230 masl	P55 across river	Single
2.	CAM 3	28.106817°	86.145039°	3835 masl	SL sight**	A pair
3.	CAM 2	28.099497°	86.137243°	3924 masl	Upper Kharka	Single
4.	CAM 9	28.118490°	86.171108°	3908 masl	Near Monastery	A pair
5.	CAM 6	28.119882°	86.178107°	4058 masl	Above monastery P58	Single
6.	CAM 5	28.128999°	86.192001°	4234 masl	Near P58	A pair
7.	CAM 10	28.093986°	86.166989°	3555 masl	MD habitat near bridge	A pair
8.	CAM 4	28.111997°	86.175000°	3842 masl	MD habitat above camp	Single
9.	CAM 7	28.041987°	86.131404°	3679 masl	Mama's hut	Single
10.	CAM 8	28.076944°	86.126031°	4255 masl	P 56 height	Single
11.	CAM 12	27.989423°	86.197338°	2883 masl	Kunzo Camp**	A pair lost
12.	Cam 13	27.968861°	86.218750°	2247 masl	GC Area**	Lost
13.	CAM 1	28.1089115 °	86.137928°	4572 masl	To Manjushree lake	A pair lost
14.	CAM 55	28.121697°	86.154420°	4230 masl	P55 across River**	Lost
15.	CAM P1	27.971464°	86.160638°	3403 masl	Samling**	Lost
16.	CAM P2	27.953008°	86.178252°	3323 masl	G_Khola**	Lost

*Note: the GPS location is taken from SW mobile app by local people, RA, and PMs.

** Cameras installed in the initial phase were lost before recording any data.

Supplementary Table S4: List of mammalian species recorded in respective camera trap

Recorded CAM no.	Assamese Monkey	Blue sheep	Himalayan Goral	Himalayan musk deer	Himalayan Serow	Himalayan Tahr	Royle's pika	Common Leopard	Himalayan Black Bear	Himalayan wolf	Leopard Cat	Red fox	Siberian weasel	Snow leopard	Yellow-throated marten	Beech marten/stone marten	Domestic dog	Domestic yak	Horse
1		✓		✓						✓	✓	✓		✓					
2		✓		✓							✓	✓						✓	
3		✓		✓			✓			✓	✓	✓		✓				✓	✓
4				✓			✓				✓	✓				✓		✓	
5		✓		✓		✓	✓			✓	✓	✓	✓	✓	✓			✓	✓
6		✓		✓		✓		✓	✓	✓	✓	✓		✓	✓			✓	✓
9		✓		✓		✓		✓	✓	✓	✓	✓	✓	✓	✓	✓	✓	✓	✓
55		✓		✓			✓			✓	✓	✓		✓				✓	✓
56						✓	✓				✓	✓		✓					
10	✓			✓	✓	✓			✓		✓	✓			✓		✓	✓	✓
11	✓		✓	✓	✓	✓		✓	✓		✓	✓			✓			✓	✓
12	✓		✓		✓	✓		✓			✓	✓			✓		✓	✓	✓
13	✓							✓				✓			✓			✓	✓
14	✓							✓				✓			✓			✓	✓
15					✓	✓		✓			✓	✓			✓			✓	✓
16					✓	✓		✓			✓	✓			✓			✓	✓
